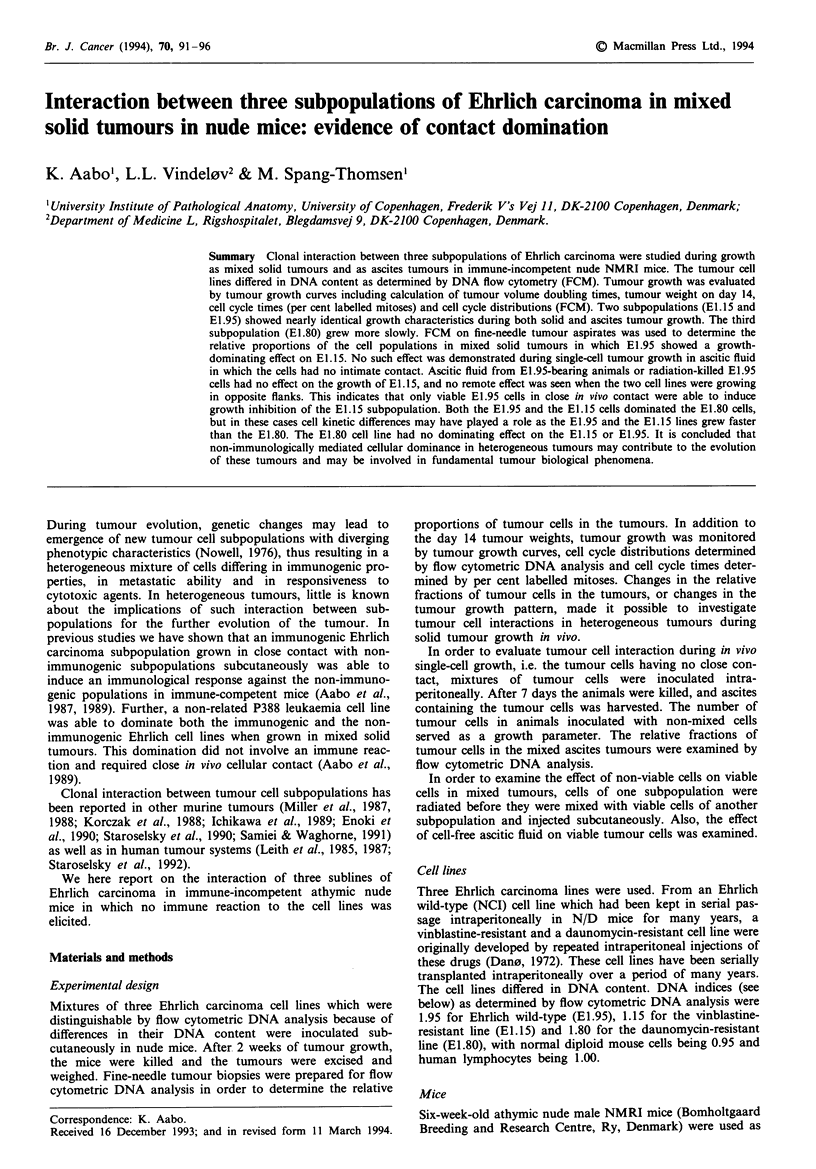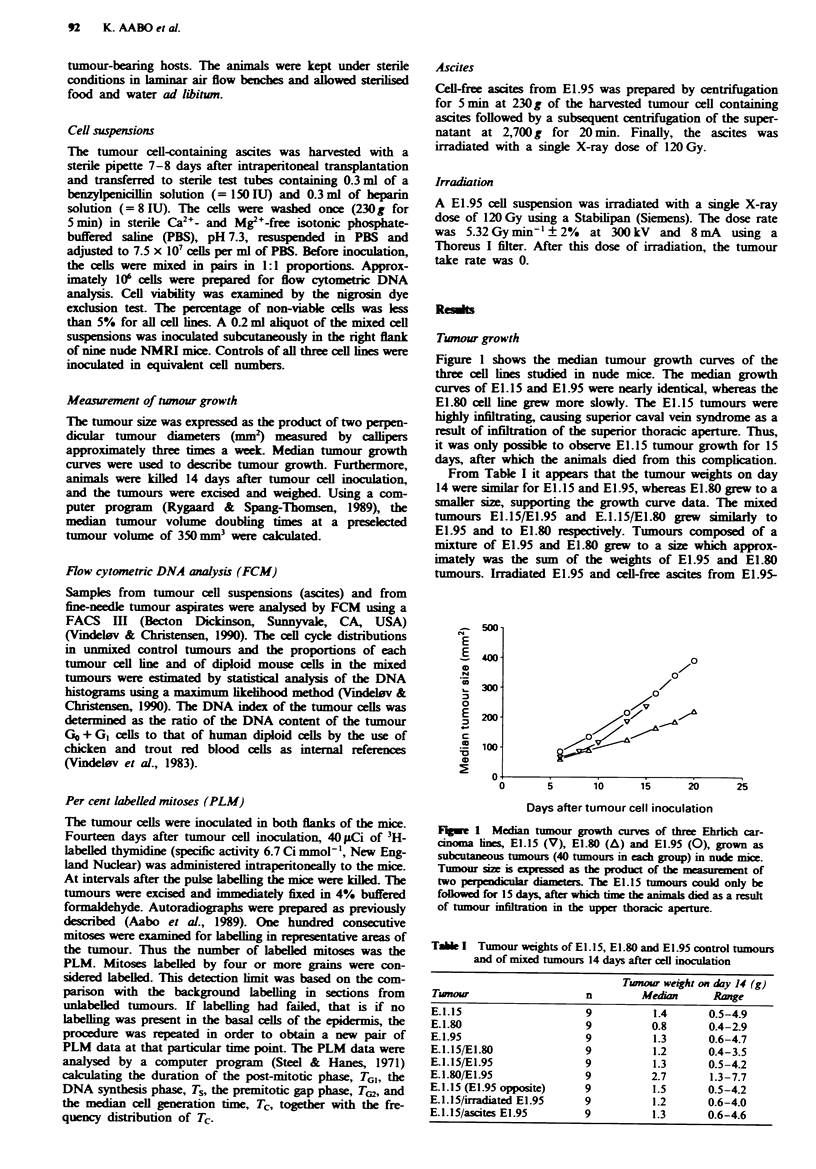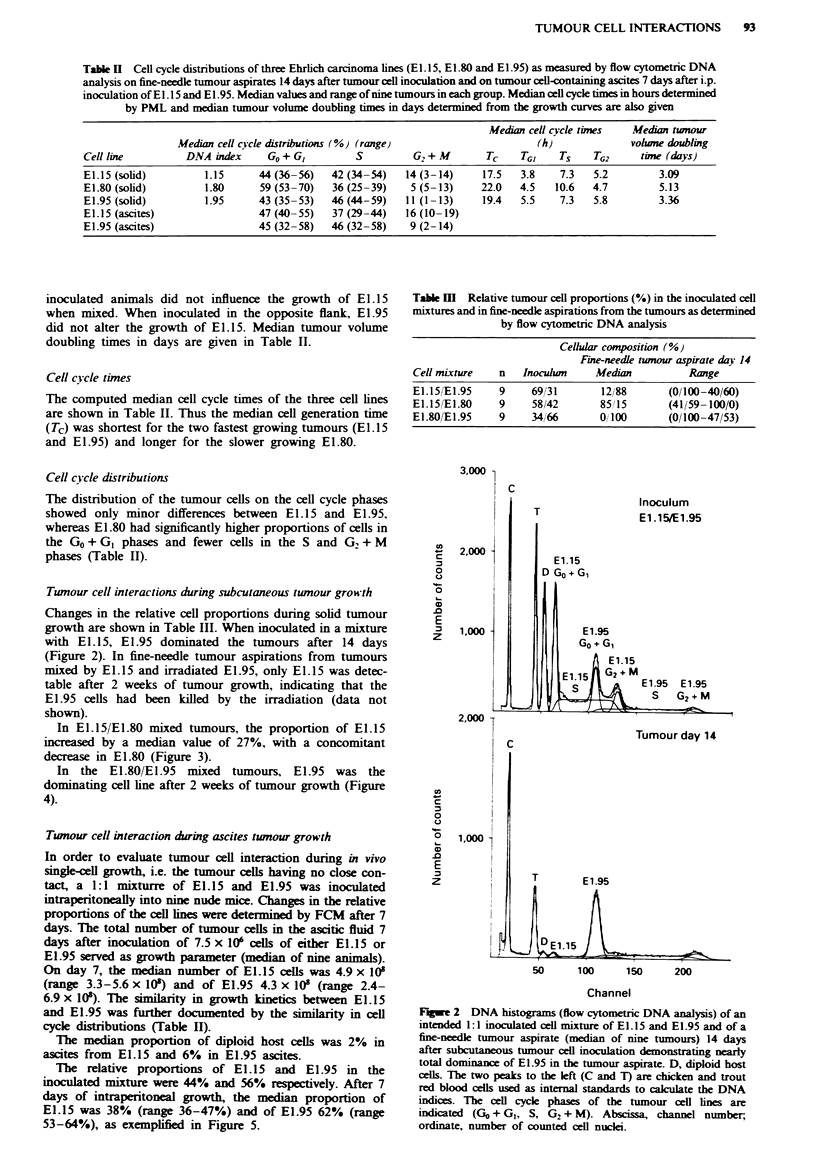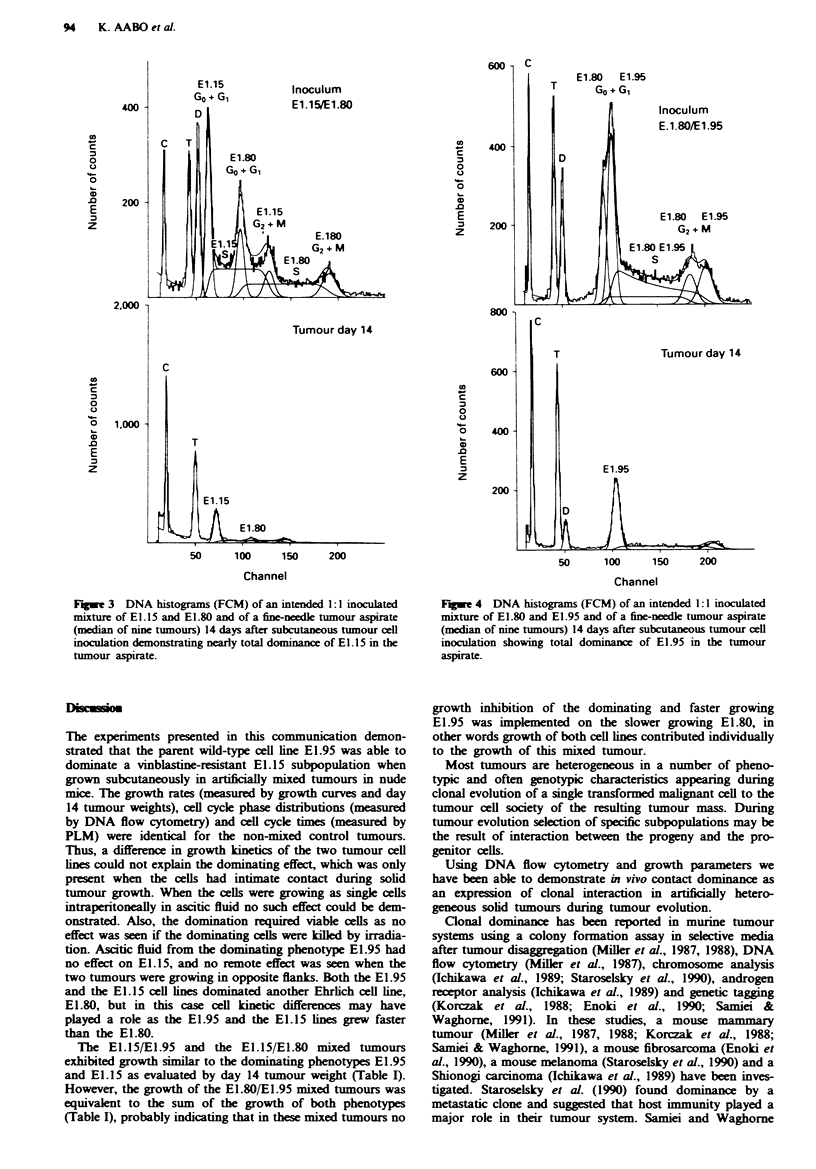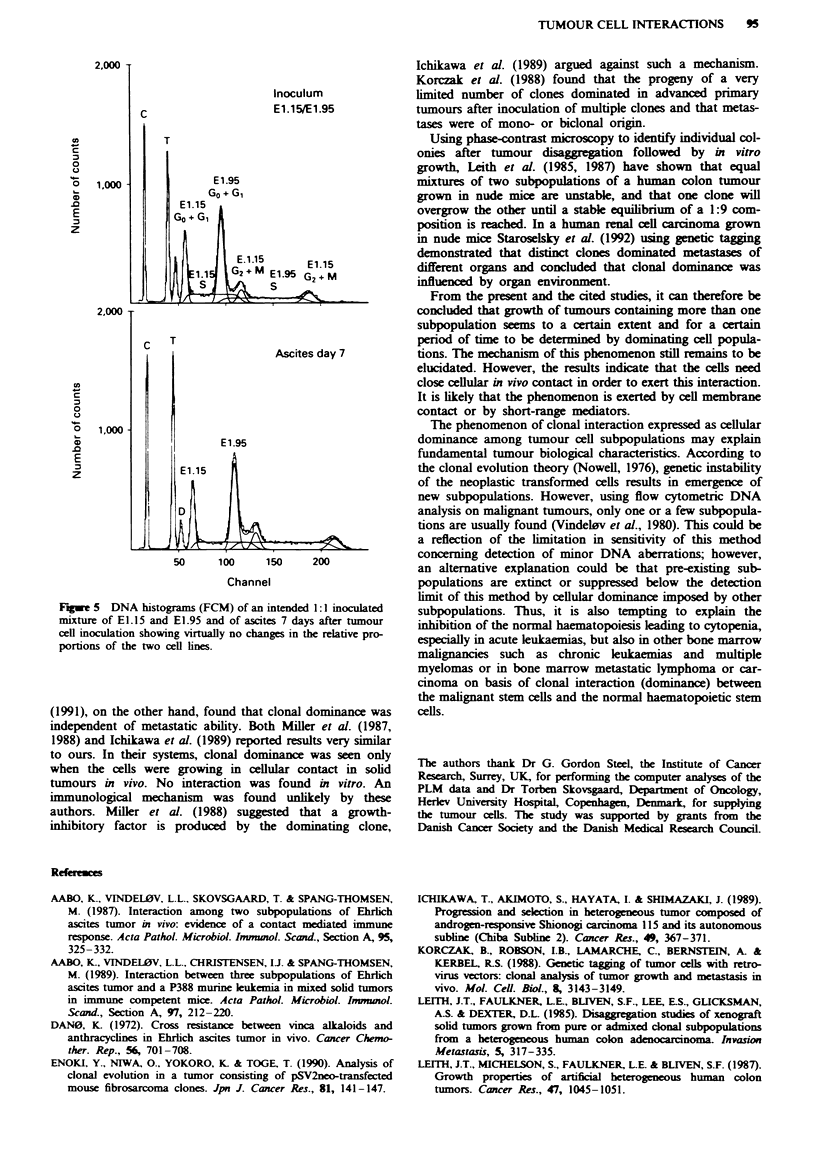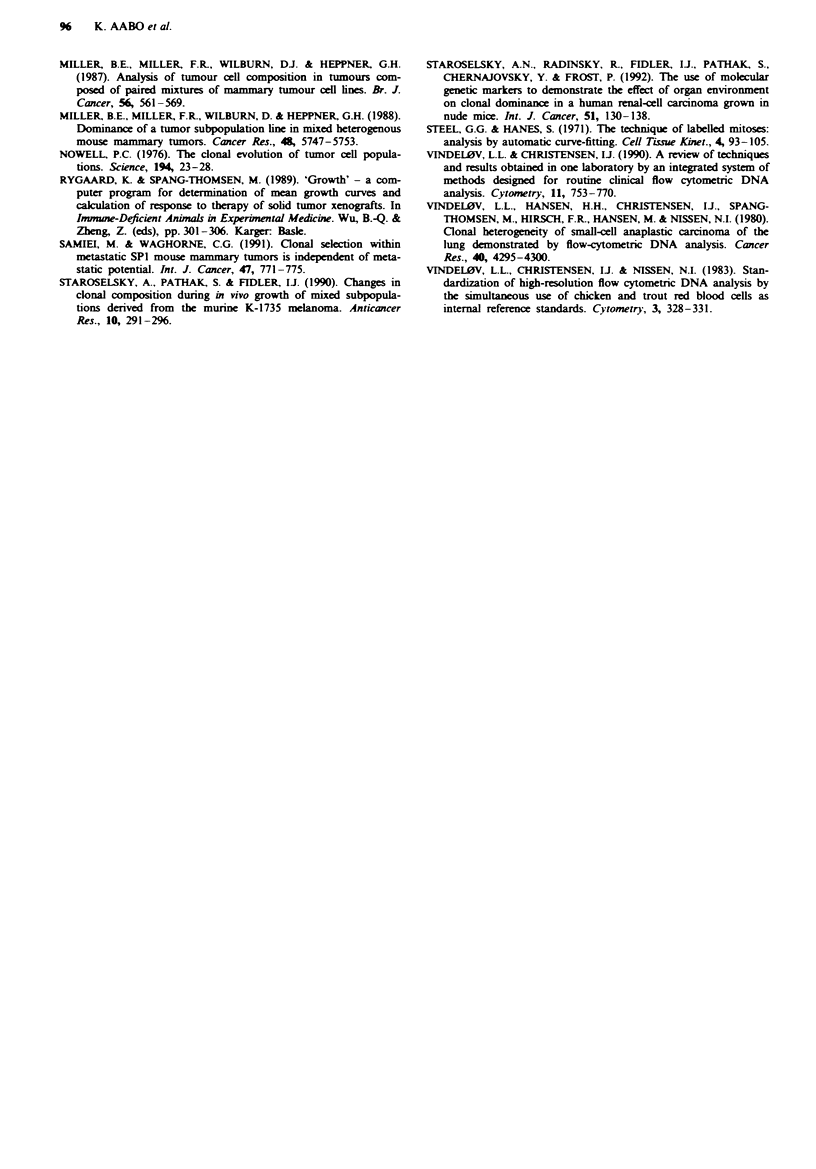# Interaction between three subpopulations of Ehrlich carcinoma in mixed solid tumours in nude mice: evidence of contact domination.

**DOI:** 10.1038/bjc.1994.255

**Published:** 1994-07

**Authors:** K. Aabo, L. L. Vindeløv, M. Spang-Thomsen

**Affiliations:** University Institute of Pathological Anatomy, University of Copenhagen, Denmark.

## Abstract

Clonal interaction between three subpopulations of Ehrlich carcinoma were studied during growth as mixed solid tumours and as ascites tumours in immune-incompetent nude NMRI mice. The tumour cell lines differed in DNA content as determined by DNA flow cytometry (FCM). Tumour growth was evaluated by tumour growth curves including calculation of tumour volume doubling times, tumour weight on day 14, cell cycle times (per cent labelled mitoses) and cell cycle distributions (FCM). Two subpopulations (E1.15 and E1.95) showed nearly identical growth characteristics during both solid and ascites tumour growth. The third subpopulation (E1.80) grew more slowly. FCM on fine-needle tumour aspirates was used to determine the relative proportions of the cell populations in mixed solid tumours in which E1.95 showed a growth-dominating effect on E1.15. No such effect was demonstrated during single-cell tumour growth in ascitic fluid in which the cells had no intimate contact. Ascitic fluid from E1.95-bearing animals or radiation-killed E1.95 cells had no effect on the growth of E1.15, and no remote effect was seen when the two cell lines were growing in opposite flanks. This indicates that only viable E1.95 cells in close in vivo contact were able to induce growth inhibition of the E1.15 subpopulation. Both the E1.95 and the E1.15 cells dominated the E1.80 cells, but in these cases cell kinetic differences may have played a role as the E1.95 and the E1.15 lines grew faster than the E1.80. The E1.80 cell line had no dominating effect on the E1.15 or E1.95. It is concluded that non-immunologically mediated cellular dominance in heterogeneous tumours may contribute to the evolution of these tumours and may be involved in fundamental tumour biological phenomena.


 © Macmillan Press Ltd., 1994

### Interaction between three subpopulations of Ehrlich carcinoma in mixed solid tumours in nude mice: evidence of contact domination

K. Aabo[1], L.L. Vindeløv[2] & M. Spang-Thomsen[1]

[1]*University Institute of Pathological Anatomy, University of Copenhagen, Frederik V's Vej 11, DK-2100 Copenhagen, Denmark;* [2]*Department of Medicine L, Rigshospitalet, Blegdamsvej 9, DK-2100 Copenhagen, Denmark.*

**Summary** Clonal interaction between three subpopulations of Ehrlich carcinoma were studied during growth as mixed solid tumours and as ascites tumours in immune-incompetent nude NMRI mice. The tumour cell lines differed in DNA content as determined by DNA flow cytometry (FCM). Tumour growth was evaluated by tumour growth curves including calculation of tumour volume doubling times, tumour weight on day 14, cell cycle times (per cent labelled mitoses) and cell cycle distributions (FCM). Two subpopulations (E1.15 and E1.95) showed nearly identical growth characteristics during both solid and ascites tumour growth. The third subpopulation (E1.80) grew more slowly. FCM on fine-needle tumour aspirates was used to determine the relative proportions of the cell populations in mixed solid tumours in which E1.95 showed a growth-dominating effect on E1.15. No such effect was demonstrated during single-cell tumour growth in ascitic fluid in which the cells had no intimate contact. Ascitic fluid from E1.95-bearing animals or radiation-killed E1.95 cells had no effect on the growth of E1.15, and no remote effect was seen when the two cell lines were growing in opposite flanks. This indicates that only viable E1.95 cells in close *in vivo* contact were able to induce growth inhibition of the E1.15 subpopulation. Both the E1.95 and the E1.15 cells dominated the E1.80 cells, but in these cases cell kinetic differences may have played a role as the E1.95 and the E1.15 lines grew faster than the E1.80. The E1.80 cell line had no dominating effect on the E1.15 or E1.95. It is concluded that non-immunologically mediated cellular dominance in heterogeneous tumours may contribute to the evolution of these tumours and may be involved in fundamental tumour biological phenomena.

During tumour evolution, genetic changes may lead to emergence of new tumour cell subpopulations with diverging phenotypic characteristics (Nowell, 1976), thus resulting in a heterogeneous mixture of cells differing in immunogenic properties, in metastatic ability and in responsiveness to cytotoxic agents. In heterogeneous tumours, little is known about the implications of such interaction between subpopulations for the further evolution of the tumour. In previous studies we have shown that an immunogenic Ehrlich carcinoma subpopulation grown in close contact with non-immunogenic subpopulations subcutaneously was able to induce an immunological response against the non-immunogenic populations in immune-competent mice (Aabo *et al.*, 1987, 1989). Further, a non-related P388 leukaemia cell line was able to dominate both the immunogenic and the non-immunogenic Ehrlich cell lines when grown in mixed solid tumours. This domination did not involve an immune reaction and required close *in vivo* cellular contact (Aabo *et al.*, 1989).

Clonal interaction between tumour cell subpopulations has been reported in other murine tumours (Miller *et al.*, 1987, 1988; Korczak *et al.*, 1988; Ichikawa *et al.*, 1989; Enoki *et al.*, 1990; Staroselsky *et al.*, 1990; Samiei & Waghorne, 1991) as well as in human tumour systems (Leith *et al.*, 1985, 1987; Staroselsky *et al.*, 1992).

We here report on the interaction of three sublines of Ehrlich carcinoma in immune-incompetent athymic nude mice in which no immune reaction to the cell lines was elicited.

### Materials and methods

### *Experimental design*

Mixtures of three Ehrlich carcinoma cell lines which were distinguishable by flow cytometric DNA analysis because of differences in their DNA content were inoculated subcutaneously in nude mice. After 2 weeks of tumour growth, the mice were killed and the tumours were excised and weighed. Fine-needle tumour biopsies were prepared for flow cytometric DNA analysis in order to determine the relative proportions of tumour cells in the tumours. In addition to the day 14 tumour weights, tumour growth was monitored by tumour growth curves, cell cycle distributions determined by flow cytometric DNA analysis and cell cycle times determined by per cent labelled mitoses. Changes in the relative fractions of tumour cells in the tumours, or changes in the tumour growth pattern, made it possible to investigate tumour cell interactions in heterogeneous tumours during solid tumour growth *in vivo*.

In order to evaluate tumour cell interaction during *in vivo* single-cell growth, i.e. the tumour cells having no close contact, mixtures of tumour cells were inoculated intraperitoneally. After 7 days the animals were killed, and ascites containing the tumour cells was harvested. The number of tumour cells in animals inoculated with non-mixed cells served as a growth parameter. The relative fractions of tumour cells in the mixed ascites tumours were examined by flow cytometric DNA analysis.

In order to examine the effect of non-viable cells on viable cells in mixed tumours, cells of one subpopulation were radiated before they were mixed with viable cells of another subpopulation and injected subcutaneously. Also, the effect of cell-free ascitic fluid on viable tumour cells was examined.

### *Cell lines*

Three Ehrlich carcinoma lines were used. From an Ehrlich wild-type (NCI) cell line which had been kept in serial passage intraperitoneally in N/D mice for many years, a vinblastine-resistant and a daunomycin-resistant cell line were originally developed by repeated intraperitoneal injections of these drugs (Danø, 1972). These cell lines have been serially transplanted intraperitoneally over a period of many years. The cell lines differed in DNA content. DNA indices (see below) as determined by flow cytometric DNA analysis were 1.95 for Ehrlich wild-type (E1.95), 1.15 for the vinblastine-resistant line (E1.15) and 1.80 for the daunomycin-resistant line (E1.80), with normal diploid mouse cells being 0.95 and human lymphocytes being 1.00.

### *Mice*

Six-week-old athymic nude male NMRI mice (Bomholtgaard Breeding and Research Centre, Ry, Denmark) were used as

Correspondence: K. Aabo.
Received 16 December 1993; and in revised form 11 March 1994.

tumour-bearing hosts. The animals were kept under sterile conditions in laminar air flow benches and allowed sterilised food and water *ad libitum*.

*Cell suspensions*

The tumour cell-containing ascites was harvested with a sterile pipette 7–8 days after intraperitoneal transplantation and transferred to sterile test tubes containing 0.3 ml of a benzylpenicillin solution (= 150 IU) and 0.3 ml of heparin solution (= 8 IU). The cells were washed once (230 *g* for 5 min) in sterile $Ca^{2+}$- and $Mg^{2+}$-free isotonic phosphate-buffered saline (PBS), pH 7.3, resuspended in PBS and adjusted to $7.5 \times 10^7$ cells per ml of PBS. Before inoculation, the cells were mixed in pairs in 1:1 proportions. Approximately $10^6$ cells were prepared for flow cytometric DNA analysis. Cell viability was examined by the nigrosin dye exclusion test. The percentage of non-viable cells was less than 5% for all cell lines. A 0.2 ml aliquot of the mixed cell suspensions was inoculated subcutaneously in the right flank of nine nude NMRI mice. Controls of all three cell lines were inoculated in equivalent cell numbers.

*Measurement of tumour growth*

The tumour size was expressed as the product of two perpendicular tumour diameters ($mm^2$) measured by callipers approximately three times a week. Median tumour growth curves were used to describe tumour growth. Furthermore, animals were killed 14 days after tumour cell inoculation, and the tumours were excised and weighed. Using a computer program (Rygaard & Spang-Thomsen, 1989), the median tumour volume doubling times at a preselected tumour volume of 350 $mm^3$ were calculated.

*Flow cytometric DNA analysis (FCM)*

Samples from tumour cell suspensions (ascites) and from fine-needle tumour aspirates were analysed by FCM using a FACS III (Becton Dickinson, Sunnyvale, CA, USA) (Vindeløv & Christensen, 1990). The cell cycle distributions in unmixed control tumours and the proportions of each tumour cell line and of diploid mouse cells in the mixed tumours were estimated by statistical analysis of the DNA histograms using a maximum likelihood method (Vindeløv & Christensen, 1990). The DNA index of the tumour cells was determined as the ratio of the DNA content of the tumour $G_0 + G_1$ cells to that of human diploid cells by the use of chicken and trout red blood cells as internal references (Vindeløv *et al.*, 1983).

*Per cent labelled mitoses (PLM)*

The tumour cells were inoculated in both flanks of the mice. Fourteen days after tumour cell inoculation, 40 µCi of $^3$H-labelled thymidine (specific activity 6.7 Ci $mmol^{-1}$, New England Nuclear) was administered intraperitoneally to the mice. At intervals after the pulse labelling the mice were killed. The tumours were excised and immediately fixed in 4% buffered formaldehyde. Autoradiographs were prepared as previously described (Aabo *et al.*, 1989). One hundred consecutive mitoses were examined for labelling in representative areas of the tumour. Thus the number of labelled mitoses was the PLM. Mitoses labelled by four or more grains were considered labelled. This detection limit was based on the comparison with the background labelling in sections from unlabelled tumours. If labelling had failed, that is if no labelling was present in the basal cells of the epidermis, the procedure was repeated in order to obtain a new pair of PLM data at that particular time point. The PLM data were analysed by a computer program (Steel & Hanes, 1971) calculating the duration of the post-mitotic phase, $T_{G1}$, the DNA synthesis phase, $T_S$, the premitotic gap phase, $T_{G2}$, and the median cell generation time, $T_C$, together with the frequency distribution of $T_C$.

*Ascites*

Cell-free ascites from E1.95 was prepared by centrifugation for 5 min at 230 *g* of the harvested tumour cell containing ascites followed by a subsequent centrifugation of the supernatant at 2,700 *g* for 20 min. Finally, the ascites was irradiated with a single X-ray dose of 120 Gy.

*Irradiation*

A E1.95 cell suspension was irradiated with a single X-ray dose of 120 Gy using a Stabilipan (Siemens). The dose rate was 5.32 Gy $min^{-1} \pm 2\%$ at 300 kV and 8 mA using a Thoreus I filter. After this dose of irradiation, the tumour take rate was 0.

### Results

*Tumour growth*

Figure 1 shows the median tumour growth curves of the three cell lines studied in nude mice. The median growth curves of E1.15 and E1.95 were nearly identical, whereas the E1.80 cell line grew more slowly. The E1.15 tumours were highly infiltrating, causing superior caval vein syndrome as a result of infiltration of the superior thoracic aperture. Thus, it was only possible to observe E1.15 tumour growth for 15 days, after which the animals died from this complication.

From Table I it appears that the tumour weights on day 14 were similar for E1.15 and E1.95, whereas E1.80 grew to a smaller size, supporting the growth curve data. The mixed tumours E1.15/E1.95 and E.1.15/E1.80 grew similarly to E1.95 and to E1.80 respectively. Tumours composed of a mixture of E1.95 and E1.80 grew to a size which approximately was the sum of the weights of E1.95 and E1.80 tumours. Irradiated E1.95 and cell-free ascites from E1.95-

**Figure 1** Median tumour growth curves of three Ehrlich carcinoma lines, E1.15 (∇), E1.80 (Δ) and E1.95 (O), grown as subcutaneous tumours (40 tumours in each group) in nude mice. Tumour size is expressed as the product of the measurement of two perpendicular diameters. The E1.15 tumours could only be followed for 15 days, after which time the animals died as a result of tumour infiltration in the upper thoracic aperture.

**Table I** Tumour weights of E1.15, E1.80 and E1.95 control tumours and of mixed tumours 14 days after cell inoculation

| *Tumour* | n | *Tumour weight on day 14 (g)* *Median* | *Range* |
|---|---|---|---|
| E.1.15 | 9 | 1.4 | 0.5–4.9 |
| E.1.80 | 9 | 0.8 | 0.4–2.9 |
| E.1.95 | 9 | 1.3 | 0.6–4.7 |
| E.1.15/E1.80 | 9 | 1.2 | 0.4–3.5 |
| E.1.15/E1.95 | 9 | 1.3 | 0.5–4.2 |
| E.1.80/E1.95 | 9 | 2.7 | 1.3–7.7 |
| E.1.15 (E1.95 opposite) | 9 | 1.5 | 0.5–4.2 |
| E.1.15/irradiated E1.95 | 9 | 1.2 | 0.6–4.0 |
| E.1.15/ascites E1.95 | 9 | 1.3 | 0.6–4.6 |

**Table II** Cell cycle distributions of three Ehrlich carcinoma lines (E1.15, E1.80 and E1.95) as measured by flow cytometric DNA analysis on fine-needle tumour aspirates 14 days after tumour cell inoculation and on tumour cell-containing ascites 7 days after i.p. inoculation of E1.15 and E1.95. Median values and range of nine tumours in each group. Median cell cycle times in hours determined by PML and median tumour volume doubling times in days determined from the growth curves are also given

| Cell line | Median cell cycle distributions (%) (range) DNA index | $G_0+G_1$ | S | $G_2+M$ | Median cell cycle times (h) $T_C$ | $T_{G1}$ | $T_S$ | $T_{G2}$ | Median tumour volume doubling time (days) |
|---|---|---|---|---|---|---|---|---|---|
| E1.15 (solid) | 1.15 | 44 (36–56) | 42 (34–54) | 14 (3–14) | 17.5 | 3.8 | 7.3 | 5.2 | 3.09 |
| E1.80 (solid) | 1.80 | 59 (53–70) | 36 (25–39) | 5 (5–13) | 22.0 | 4.5 | 10.6 | 4.7 | 5.13 |
| E1.95 (solid) | 1.95 | 43 (35–53) | 46 (44–59) | 11 (1–13) | 19.4 | 5.5 | 7.3 | 5.8 | 3.36 |
| E1.15 (ascites) | | 47 (40–55) | 37 (29–44) | 16 (10–19) | | | | | |
| E1.95 (ascites) | | 45 (32–58) | 46 (32–58) | 9 (2–14) | | | | | |

inoculated animals did not influence the growth of E1.15 when mixed. When inoculated in the opposite flank, E1.95 did not alter the growth of E1.15. Median tumour volume doubling times in days are given in Table II.

### *Cell cycle times*

The computed median cell cycle times of the three cell lines are shown in Table II. Thus the median cell generation time ($T_C$) was shortest for the two fastest growing tumours (E1.15 and E1.95) and longer for the slower growing E1.80.

### *Cell cycle distributions*

The distribution of the tumour cells on the cell cycle phases showed only minor differences between E1.15 and E1.95, whereas E1.80 had significantly higher proportions of cells in the $G_0+G_1$ phases and fewer cells in the S and $G_2+M$ phases (Table II).

### *Tumour cell interactions during subcutaneous tumour growth*

Changes in the relative cell proportions during solid tumour growth are shown in Table III. When inoculated in a mixture with E1.15, E1.95 dominated the tumours after 14 days (Figure 2). In fine-needle tumour aspirations from tumours mixed by E1.15 and irradiated E1.95, only E1.15 was detectable after 2 weeks of tumour growth, indicating that the E1.95 cells had been killed by the irradiation (data not shown).

In E1.15/E1.80 mixed tumours, the proportion of E1.15 increased by a median value of 27%, with a concomitant decrease in E1.80 (Figure 3).

In the E1.80/E1.95 mixed tumours, E1.95 was the dominating cell line after 2 weeks of tumour growth (Figure 4).

### *Tumour cell interaction during ascites tumour growth*

In order to evaluate tumour cell interaction during *in vivo* single-cell growth, i.e. the tumour cells having no close contact, a 1:1 mixturre of E1.15 and E1.95 was inoculated intraperitoneally into nine nude mice. Changes in the relative proportions of the cell lines were determined by FCM after 7 days. The total number of tumour cells in the ascitic fluid 7 days after inoculation of $7.5 \times 10^6$ cells of either E1.15 or E1.95 served as growth parameter (median of nine animals). On day 7, the median number of E1.15 cells was $4.9 \times 10^8$ (range $3.3-5.6 \times 10^8$) and of E1.95 $4.3 \times 10^8$ (range $2.4-6.9 \times 10^8$). The similarity in growth kinetics between E1.15 and E1.95 was further documented by the similarity in cell cycle distributions (Table II).

The median proportion of diploid host cells was 2% in ascites from E1.15 and 6% in E1.95 ascites.

The relative proportions of E1.15 and E1.95 in the inoculated mixture were 44% and 56% respectively. After 7 days of intraperitoneal growth, the median proportion of E1.15 was 38% (range 36–47%) and of E1.95 62% (range 53–64%), as exemplified in Figure 5.

**Table III** Relative tumour cell proportions (%) in the inoculated cell mixtures and in fine-needle aspirations from the tumours as determined by flow cytometric DNA analysis

| Cell mixture | n | Cellular composition (%) Inoculum | Fine-needle tumour aspirate day 14 Median | Range |
|---|---|---|---|---|
| E1.15/E1.95 | 9 | 69/31 | 12/88 | (0/100–40/60) |
| E1.15/E1.80 | 9 | 58/42 | 85/15 | (41/59–100/0) |
| E1.80/E1.95 | 9 | 34/66 | 0/100 | (0/100–47/53) |

**Figure 2** DNA histograms (flow cytometric DNA analysis) of an intended 1:1 inoculated cell mixture of E1.15 and E1.95 and of a fine-needle tumour aspirate (median of nine tumours) 14 days after subcutaneous tumour cell inoculation demonstrating nearly total dominance of E1.95 in the tumour aspirate. D, diploid host cells. The two peaks to the left (C and T) are chicken and trout red blood cells used as internal standards to calculate the DNA indices. The cell cycle phases of the tumour cell lines are indicated ($G_0+G_1$, S, $G_2+M$). Abscissa, channel number; ordinate, number of counted cell nuclei.

Figure 3 DNA histograms (FCM) of an intended 1:1 inoculated mixture of E1.15 and E1.80 and of a fine-needle tumour aspirate (median of nine tumours) 14 days after subcutaneous tumour cell inoculation demonstrating nearly total dominance of E1.15 in the tumour aspirate.

Figure 4 DNA histograms (FCM) of an intended 1:1 inoculated mixture of E1.80 and E1.95 and of a fine-needle tumour aspirate (median of nine tumours) 14 days after subcutaneous tumour cell inoculation showing total dominance of E1.95 in the tumour aspirate.

### Discussion

The experiments presented in this communication demonstrated that the parent wild-type cell line E1.95 was able to dominate a vinblastine-resistant E1.15 subpopulation when grown subcutaneously in artificially mixed tumours in nude mice. The growth rates (measured by growth curves and day 14 tumour weights), cell cycle phase distributions (measured by DNA flow cytometry) and cell cycle times (measured by PLM) were identical for the non-mixed control tumours. Thus, a difference in growth kinetics of the two tumour cell lines could not explain the dominating effect, which was only present when the cells had intimate contact during solid tumour growth. When the cells were growing as single cells intraperitoneally in ascitic fluid no such effect could be demonstrated. Also, the domination required viable cells as no effect was seen if the dominating cells were killed by irradiation. Ascitic fluid from the dominating phenotype E1.95 had no effect on E1.15, and no remote effect was seen when the two tumours were growing in opposite flanks. Both the E1.95 and the E1.15 cell lines dominated another Ehrlich cell line, E1.80, but in this case cell kinetic differences may have played a role as the E1.95 and the E1.15 lines grew faster than the E1.80.

The E1.15/E1.95 and the E1.15/E1.80 mixed tumours exhibited growth similar to the dominating phenotypes E1.95 and E1.15 as evaluated by day 14 tumour weight (Table I). However, the growth of the E1.80/E1.95 mixed tumours was equivalent to the sum of the growth of both phenotypes (Table I), probably indicating that in these mixed tumours no growth inhibition of the dominating and faster growing E1.95 was implemented on the slower growing E1.80, in other words growth of both cell lines contributed individually to the growth of this mixed tumour.

Most tumours are heterogeneous in a number of phenotypic and often genotypic characteristics appearing during clonal evolution of a single transformed malignant cell to the tumour cell society of the resulting tumour mass. During tumour evolution selection of specific subpopulations may be the result of interaction between the progeny and the progenitor cells.

Using DNA flow cytometry and growth parameters we have been able to demonstrate *in vivo* contact dominance as an expression of clonal interaction in artificially heterogeneous solid tumours during tumour evolution.

Clonal dominance has been reported in murine tumour systems using a colony formation assay in selective media after tumour disaggregation (Miller *et al.*, 1987, 1988), DNA flow cytometry (Miller *et al.*, 1987), chromosome analysis (Ichikawa *et al.*, 1989; Staroselsky *et al.*, 1990), androgen receptor analysis (Ichikawa *et al.*, 1989) and genetic tagging (Korczak *et al.*, 1988; Enoki *et al.*, 1990; Samiei & Waghorne, 1991). In these studies, a mouse mammary tumour (Miller *et al.*, 1987, 1988; Korczak *et al.*, 1988; Samiei & Waghorne, 1991), a mouse fibrosarcoma (Enoki *et al.*, 1990), a mouse melanoma (Staroselsky *et al.*, 1990) and a Shionogi carcinoma (Ichikawa *et al.*, 1989) have been investigated. Staroselsky *et al.* (1990) found dominance by a metastatic clone and suggested that host immunity played a major role in their tumour system. Samiei and Waghorne

**Figure 5** DNA histograms (FCM) of an intended 1:1 inoculated mixture of E1.15 and E1.95 and of ascites 7 days after tumour cell inoculation showing virtually no changes in the relative proportions of the two cell lines.

(1991), on the other hand, found that clonal dominance was independent of metastatic ability. Both Miller *et al.* (1987, 1988) and Ichikawa *et al.* (1989) reported results very similar to ours. In their systems, clonal dominance was seen only when the cells were growing in cellular contact in solid tumours *in vivo*. No interaction was found *in vitro*. An immunological mechanism was found unlikely by these authors. Miller *et al.* (1988) suggested that a growth-inhibitory factor is produced by the dominating clone, Ichikawa *et al.* (1989) argued against such a mechanism. Korczak *et al.* (1988) found that the progeny of a very limited number of clones dominated in advanced primary tumours after inoculation of multiple clones and that metastases were of mono- or biclonal origin.

Using phase-contrast microscopy to identify individual colonies after tumour disaggregation followed by *in vitro* growth, Leith *et al.* (1985, 1987) have shown that equal mixtures of two subpopulations of a human colon tumour grown in nude mice are unstable, and that one clone will overgrow the other until a stable equilibrium of a 1:9 composition is reached. In a human renal cell carcinoma grown in nude mice Staroselsky *et al.* (1992) using genetic tagging demonstrated that distinct clones dominated metastases of different organs and concluded that clonal dominance was influenced by organ environment.

From the present and the cited studies, it can therefore be concluded that growth of tumours containing more than one subpopulation seems to a certain extent and for a certain period of time to be determined by dominating cell populations. The mechanism of this phenomenon still remains to be elucidated. However, the results indicate that the cells need close cellular *in vivo* contact in order to exert this interaction. It is likely that the phenomenon is exerted by cell membrane contact or by short-range mediators.

The phenomenon of clonal interaction expressed as cellular dominance among tumour cell subpopulations may explain fundamental tumour biological characteristics. According to the clonal evolution theory (Nowell, 1976), genetic instability of the neoplastic transformed cells results in emergence of new subpopulations. However, using flow cytometric DNA analysis on malignant tumours, only one or a few subpopulations are usually found (Vindeløv *et al.*, 1980). This could be a reflection of the limitation in sensitivity of this method concerning detection of minor DNA aberrations; however, an alternative explanation could be that pre-existing subpopulations are extinct or suppressed below the detection limit of this method by cellular dominance imposed by other subpopulations. Thus, it is also tempting to explain the inhibition of the normal haematopoiesis leading to cytopenia, especially in acute leukaemias, but also in other bone marrow malignancies such as chronic leukaemias and multiple myelomas or in bone marrow metastatic lymphoma or carcinoma on basis of clonal interaction (dominance) between the malignant stem cells and the normal haematopoietic stem cells.

The authors thank Dr G. Gordon Steel, the Institute of Cancer Research, Surrey, UK, for performing the computer analyses of the PLM data and Dr Torben Skovsgaard, Department of Oncology, Herlev University Hospital, Copenhagen, Denmark, for supplying the tumour cells. The study was supported by grants from the Danish Cancer Society and the Danish Medical Research Council.

### References

AABO, K., VINDELØV, L.L., SKOVSGAARD, T. & SPANG-THOMSEN, M. (1987). Interaction among two subpopulations of Ehrlich ascites tumor *in vivo*: evidence of a contact mediated immune response. *Acta Pathol. Microbiol. Immunol. Scand.*, Section A, **95**, 325–332.

AABO, K., VINDELØV, L.L., CHRISTENSEN, I.J. & SPANG-THOMSEN, M. (1989). Interaction between three subpopulations of Ehrlich ascites tumor and a P388 murine leukemia in mixed solid tumors in immune competent mice. *Acta Pathol. Microbiol. Immunol. Scand.*, Section A, **97**, 212–220.

DANØ, K. (1972). Cross resistance between vinca alkaloids and anthracyclines in Ehrlich ascites tumor in vivo. *Cancer Chemother. Rep.*, **56**, 701–708.

ENOKI, Y., NIWA, O., YOKORO, K. & TOGE, T. (1990). Analysis of clonal evolution in a tumor consisting of pSV2neo-transfected mouse fibrosarcoma clones. *Jpn J. Cancer Res.*, **81**, 141–147.

ICHIKAWA, T., AKIMOTO, S., HAYATA, I. & SHIMAZAKI, J. (1989). Progression and selection in heterogeneous tumor composed of androgen-responsive Shionogi carcinoma 115 and its autonomous subline (Chiba Subline 2). *Cancer Res.*, **49**, 367–371.

KORCZAK, B., ROBSON, I.B., LAMARCHE, C., BERNSTEIN, A. & KERBEL, R.S. (1988). Genetic tagging of tumor cells with retrovirus vectors: clonal analysis of tumor growth and metastasis in vivo. *Mol. Cell. Biol.*, **8**, 3143–3149.

LEITH, J.T., FAULKNER, L.E., BLIVEN, S.F., LEE, E.S., GLICKSMAN, A.S. & DEXTER, D.L. (1985). Disaggregation studies of xenograft solid tumors grown from pure or admixed clonal subpopulations from a heterogeneous human colon adenocarcinoma. *Invasion Metastasis*, **5**, 317–335.

LEITH, J.T., MICHELSON, S., FAULKNER, L.E. & BLIVEN, S.F. (1987). Growth properties of artificial heterogeneous human colon tumors. *Cancer Res.*, **47**, 1045–1051.

MILLER, B.E., MILLER, F.R., WILBURN, D.J. & HEPPNER, G.H. (1987). Analysis of tumour cell composition in tumours composed of paired mixtures of mammary tumour cell lines. *Br. J. Cancer*, **56**, 561-569.

MILLER, B.E., MILLER, F.R., WILBURN, D. & HEPPNER, G.H. (1988). Dominance of a tumor subpopulation line in mixed heterogenous mouse mammary tumors. *Cancer Res.*, **48**, 5747-5753.

NOWELL, P.C. (1976). The clonal evolution of tumor cell populations. *Science*, **194**, 23-28.

RYGAARD, K. & SPANG-THOMSEN, M. (1989). 'Growth' - a computer program for determination of mean growth curves and calculation of response to therapy of solid tumor xenografts. In *Immune-Deficient Animals in Experimental Medicine*. Wu, B.-Q. & Zheng, Z. (eds), pp. 301-306. Karger: Basle.

SAMIEI, M. & WAGHORNE, C.G. (1991). Clonal selection within metastatic SP1 mouse mammary tumors is independent of metastatic potential. *Int. J. Cancer*, **47**, 771-775.

STAROSELSKY, A., PATHAK, S. & FIDLER, I.J. (1990). Changes in clonal composition during *in vivo* growth of mixed subpopulations derived from the murine K-1735 melanoma. *Anticancer Res.*, **10**, 291-296.

STAROSELSKY, A.N., RADINSKY, R., FIDLER, I.J., PATHAK, S., CHERNAJOVSKY, Y. & FROST, P. (1992). The use of molecular genetic markers to demonstrate the effect of organ environment on clonal dominance in a human renal-cell carcinoma grown in nude mice. *Int. J. Cancer*, **51**, 130-138.

STEEL, G.G. & HANES, S. (1971). The technique of labelled mitoses: analysis by automatic curve-fitting. *Cell Tissue Kinet.*, **4**, 93-105.

VINDELØV, L.L. & CHRISTENSEN, I.J. (1990). A review of techniques and results obtained in one laboratory by an integrated system of methods designed for routine clinical flow cytometric DNA analysis. *Cytometry*, **11**, 753-770.

VINDELØV, L.L., HANSEN, H.H., CHRISTENSEN, I.J., SPANG-THOMSEN, M., HIRSCH, F.R., HANSEN, M. & NISSEN, N.I. (1980). Clonal heterogeneity of small-cell anaplastic carcinoma of the lung demonstrated by flow-cytometric DNA analysis. *Cancer Res.*, **40**, 4295-4300.

VINDELØV, L.L., CHRISTENSEN, I.J. & NISSEN, N.I. (1983). Standardization of high-resolution flow cytometric DNA analysis by the simultaneous use of chicken and trout red blood cells as internal reference standards. *Cytometry*, **3**, 328-331.